# The effect of educational intervention through sending emails on improving physical posture in female computer users of Eastern Iran: a quasi-experiment study

**DOI:** 10.3389/fdgth.2024.1427693

**Published:** 2024-12-10

**Authors:** Zahra Hosseini, Arash Ziapour, Seyyede Fateme Rahimi, Fatemeh Dalake, Murat Yıldırım

**Affiliations:** ^1^ Social Determinants in Health Promotion Research Center, Hormozgan Health Institute, Hormozgan University of Medical Sciences, Bandar Abbas, Iran; ^2^ Cardiovascular Research Center, Health Policy and Promotion Institute, Kermanshah University of Medical Sciences, Kermanshah, Iran; ^3^ Psychology Research Centre, Khazar University, Baku, Azerbaijan; ^4^ Student Research Committee, Hormozgan University of Medical Sciences, Bandar Abbas, Iran; ^5^ Department of Occupational Health Engineering, Birjand University of Medical Sciences, Birjand, Iran; ^6^ Department of Psychology, Faculty of Science and Letters, Agri Ibrahim Cecen University, Agri, Türkiye

**Keywords:** training, email, computer users, ergonomics, women, Iran, working, posture

## Abstract

**Background:**

Musculoskeletal disorders are among the most common occupational injuries and disabilities in developing and industrialized countries. This study aims to determine the effectiveness of e-mail training to improve the physical posture of female computer users at Birjand University of Medical Sciences in Iran.

**Methods:**

The present interventional research explores the effect of email-based training to correct the body posture of female computer users in Birjand, Iran. In this quasi-experiment, 120 women who worked in Birjand University of Medical Sciences using computers were selected through a census. 60 computer users were selected from the deputy of education and 60 from the deputy of development for the intervention group (IG) and control group (CG), respectively. A training program was developed on the ergonomics of office work (12 emails at an interval of 6 weeks). The data was collected using demographic, occupational information, and a knowledge assessment questionnaire. Nordic Musculoskeletal Questionnaire (NMQ) and Rapid Office Strain Assessment (ROSA) were used in both groups before the intervention and 6 months later.

**Results:**

After the educational intervention, a significant increase was observed in the ergonomics knowledge of the IG compared to the control. The ROSA score was lowered from a high-risk to a low-and medium-risk level (*p* < 0.05). In the IG, 44 subjects (73.30%) who needed ergonomic intervention (a score above 5) were reduced to 10 subjects (16.70%) with a need for ergonomic intervention. According to NMQ, the highest frequency of pain in the IG and CG was related to the back (56.70% and 55%, respectively). The neck, shoulders, wrists, back and elbows were next.

**Conclusions:**

This quasi-intervention study was conducted to determine the effect of email-based training on correcting female computer users’ body posture in 2022. Training ergonomics through email is a practical and acceptable way to improve ergonomic behaviors among computer users. It enables them to adapt to the workplace by applying the correct ergonomics, changing their work behavior to prevent occupational musculoskeletal disorders, and reduce risks and complications.

## 1 Introduction

Musculoskeletal disorders include pressure and pain in different parts of the body such as the wrists, elbows, neck, and shoulders. These disorders also include symptoms of severe muscle fatigue and pain, especially in the back and lower back (1, 2). This can be due to several risk factors related to workplace and physical activity involving improper ergonomic positions for an extended period of time (3). Musculoskeletal disorders are the main cause of disability around the world (4). In Iran, it is the fourth leading cause of disability (5). Work-related musculoskeletal disorders are considered an integral problem associated with health, disability, and absenteeism; it is the main cause of damage to the workforce (6). The diseases caused by these disorders not only affect the individual's quality of life but also impose a great economic burden on society (3, 7, 8).

Numerous jobs are linked to musculoskeletal disorders. Scientific reports suggest that those who use computers for long hours have a higher risk of developing these problems (9, 10). Excessive use of computers is associated with pain in the back, neck, shoulders, elbows, and wrist (11–13). The major causes of musculoskeletal disorders in computer users are repetitive activity, excessive force, inappropriate posture, contact pressure, vibration, and physical fatigue (14). The prevalence rate of musculoskeletal symptoms in computer users is reported to be 50% (9). In a study, the prevalence of musculoskeletal disorders in computer users was estimated to be 67.9%, which occurred more in the neck and shoulders. Also, this study showed that women are 2.059 times more likely to show symptoms than men (15). Studies conducted in Iran have shown that 87% of the official personnel of Guilan University of Medical Sciences complained of the symptoms (16). Moreover, among the personnel of the Tehran University of Medical Sciences, more than 90% of computer users did not observe the correct principles of working with computers. In this regard, the prevalence of musculoskeletal symptoms was 59.7% in the neck, 42% in the shoulder, 41.5% in the waist, and 39.2% in the back (17).

An effective method to estimate the risks of musculoskeletal injuries in office tasks and computer work is ROSA, the Rapid Administrative Stress Assessment, published during 2011–2012 by Sonne et al. at the University of Windsor, Canada (18). The aim of ROSA was to offer a pragmatic approach for taking into account the key aspects from an ergonomic perspective (seating, workstations, etc.) within a company or workplace. In this method, the posture of the personnel while working is analyzed using an observation method and a checklist. ROSA was developed out of the previous assessment methods and added more focus on office workers, especially those working with computers. This method is based on CSA standard Z412, a process-oriented document and a step-by-step guideline for the inclusion and implementation of ergonomics for design and layout of office jobs, work organization, environmental conditions, and workstation design. It is also based on CSA standard EN-ISO 9241, 1997, which is a standard framework for the measurement of ergonomic problems. This method is also reliable and valid in measuring the ergonomic risk factors in office work with computers included (19). The prevalence of musculoskeletal disorders in the workplace is directly related to ergonomics in the workplace such as repetitive movements, inappropriate posture, and excessive force. Inappropriate posture at work is a major risk factor for these disorders (20, 21). There are many reasons that ergonomics are not observed by computer users in the workplace, including a lack of knowledge (9). Attempting to increase knowledge and change personnel attitude can be effective in creating a safe workplace and culture (22). According to ergonomics training research, this method can act as an effective strategy in improving computer users knowledge of risk factors (23), reducing musculoskeletal injuries (24), and improving users’ posture and workstation layout (23).

In the current era, most educational research activities are performed in cyberspace (25). E-learning is a type of multimedia training that improves human resources. Its advantages include learning anywhere and anytime, inherent flexibility, and having a lower cost than in-person learning (26). E-learning provides a unique experience by using three conventions, visual, auditory, and textual modes, to teach simultaneously. E-learning also provides private training and allows people to determine the rate of academic progress at will, matching with their learning ability (27). Women comprise a large portion of university personnel that use the computer for long, consecutive hours. Data reveals that they spend their daily time in a constant posture, suffering a high prevalence of musculoskeletal disorders as computer users. Ergonomic interventions, presentation, and implementation of control strategies to correct the posture and reduce musculoskeletal injuries are needed, especially for this demographic. Also, it should be taken into account the increasing expansion of E-learning due to the features of multimedia training (28) Sending E-learning packages to computer users who access the Internet is free of charge (29). The related literature reported the high prevalence of musculoskeletal disorders in women compared to men (30). We have several goals from this study. First, we want to define the level of ergonomic knowledge of our target group. Second, we want to investigate the prevalence of musculoskeletal disorders in the target group and the ergonomic status of computer users in the study sample. Last, we want to determine the effect of the educational intervention on improving the physical condition of the target group.

## 2 Materials and methods

### 2.1 Research design and participation

This quasi-intervention study was conducted to determine the effect of email-based training on correcting female computer users’ body posture in 2022. The study population consists of women working in Birjand University of Medical Sciences, Iran. To prevent the exchange of information between the Intervention group and control group, the samples were selected from the Deputy of Education as well as the Deputy of Development at Birjand University of Medical Sciences. The physical work environments of the employees are identical in terms of physical facilities, however they are located at an appropriate distance, in two separate buildings in the university.

### 2.2 Sampling and randomization

One deputy was considered as the IG and the other as the CG at random. The sample size was 120 to include all women working in the deputy of Education and Development based on the inclusion criteria (60 computer users in the deputy of Education and 60 computer users in the deputy of Development in the IG and CG, respectively).

### 2.3 Inclusion criteria and exclusion criteria

The inclusion criteria consist of having at least one year of experience working with a computer, working with a computer for more than 20 h per week, no history of accidents or diseases affecting the musculoskeletal system (lupus, osteoarthritis, gout, rheumatoid arthritis, diabetes, and thyroid), not being pregnant, not consuming any painkillers, and not passing any ergonomics course for working with computers. The exclusion criteria also included dissatisfaction with participating in the study, non-cooperation in training sessions or reading emails, and having a history of severe trauma or fracture in the neck, elbow, back, or arms.

### 2.4 Intervention

After completing the questionnaires and preliminary data analysis regarding ergonomics and Nordic knowledge scores, a training program was developed in collaboration with occupational health engineers, tailored to the needs of the target group. The goal of this program was to enhance the knowledge of ergonomics, increase the ROSA score, and improve the physical postures of computer users. The educational content included a short instructional video and PowerPoint presentations with a small volume and brief duration to ensure it did not take up much time for individuals. This content focused on familiarizing participants with skeletal and muscular disorders caused by improper computer use and optimizing conditions for equipment usage.

The training was delivered in 12 emails over a 6-week period, with two emails sent each week. All participants in the intervention group joined a WhatsApp group, which notified them when a new email was sent. If participants had questions or doubts about the educational content, these were raised and addressed within the same group. One of the researchers was responsible for asking the subjects after each email was sent whether they had received the educational content. After 6 months, the intervention test was conducted.

### 2.5 Data collection tools

#### 2.5.1 Standard nordic musculoskeletal problems questionnaire

The Nordic Standard Musculoskeletal Problems Questionnaire is one of the most common questionnaires for determining the signs and symptoms of musculoskeletal disorders, developed by Corina et al. Since 1987, the necessary information for gathering data on musculoskeletal disorders, and obtaining information on disease prevalence and epidemiology, were collected using this questionnaire as a standard approach (31). The Nordic questionnaire was used to determine the prevalence of musculoskeletal disorders in 9 areas of the body (neck, shoulders, back, waist, elbows, hands, wrists, thighs, legs, and ankles) (32). This questionnaire has 40 items. Respondents were asked if they had any musculoskeletal trouble in the last 12 months and last 7 days that prevented normal activity. Comparing pain has sensitivity ranged between 66 and 92% and specificity between 71 and 88% (33). The validity and reliability of this questionnaire has been confirmed by Mokhtarnia et al. for the use of Iranian users (34).

#### 2.5.2 Rapid administrative stress assessment (ROSA) checklist

The Rapid Administrative Stress Assessment (ROSA) checklist was used to determine risk factors and risk levels. This method was published by Sonne et al. at the University of Windsor, Canada, from 2011 to 2012, to rapidly determine the risks of musculoskeletal injuries related to office and computer tasks (18). The purpose of designing this method was to create a practical tool for identifying important areas in an organization or office from an ergonomic point of view. In this method, the physical condition of individuals while performing administrative tasks is analyzed using the observation method and checklist. By building upon its previous assessment methods and placing greater emphasis on the activities of office users, particularly those working with computers based on CSA standard Z412 and EN-ISO 9241, 1997, the Rapid Office Stress Assessment (ROSA) method was developed. This method also has high validity and reliability in measuring the risk of ergonomic factors in the office environment and while working with computers (19).

In evaluating ergonomic risk factors, the workstation is divided into several sections. Sections include seat components, monitors, telephones, mice, and keyboards. The risk level of each section is determined through an evaluation process consisting of three main parts. In the first part, the physical condition of individuals in a sitting position is evaluated according to the position of the thighs, knees, elbows, and waist. This evaluation is based on the position of the height of the chair, the depth of the seat, the position of the armrest, and the backrest. In the second part, the condition of the neck and shoulders is assessed based on the height of the screen and access to the phone. In the third part, the condition of the wrist will be analyzed based on working with the mouse and keyboard, and the risk will be scored. After coding the risk factors identified in each section, neutral postures received a minimum score of 1, and deviation from these postures received a score of 1–3. The posture establishment time was added to the above score according to the checklist. The duration of the postures will be calculated in 3 positions, which are: position A related to the height of the chair, the depth of the seat, the armrest, and the backrest. In this section, points related to the chair are obtained based on the duration of using the chair. In Section B, monitor position and telephone position scores are obtained based on the appropriate duration. In section C, the points obtained from working with the mouse and keyboard are obtained according to the duration of working with these devices. The scores from sections C and B represent the scores of the monitor and accessories. After completing each section and determining the scores, the final ROSA score was determined and the score obtained from this method was measured before and after the study. The final score range resulting from the examined risk factors falls between 0 and 10, with scores of 0 to 5 representing low and medium risk levels, and scores higher than 5 being categorized as requiring intervention (18). Questionnaires were completed by both experimental and control groups before and 6 months after the intervention.

### 2.6 Statistical analysis

Data analysis was conducted using SPSS 22 software with a significance level of 0.05. Quantitative data was described using mean and standard deviation, while qualitative data was described using frequency and percentage. Additionally, statistical tests such as independent *t*-test, paired *t*-test, chi-square and Anova were utilized. First, the normality of quantitative data was checked using the Kolmogorov-Smirnov test. The Significance level was considered 0.05 for all tests.

### 2.7 Ethics approval and consent to participate

First, the required permissions were gained from the Research Deputy. We were referred to the Deputy of Education and Development of Birjand University of Medical Sciences. We used the inclusion criteria to select the sample. After explaining the objectives of the study and getting informed written consent, the questionnaires were provided to the participants. This project has been approved with a code of ethics (#IR.HUMS.REC.1398.432) in Hormozgan University of Medical Sciences.

## 3 Results

The total number of employees in the two departments was 154 people, 73 people in the Education Department and 81 people in the Development Department. We included 120 women in the study by census method and based on entry and exit criteria, 60 people from the Education Department and 60 people from the Development Department. To describe research sample, we compared two groups in terms of quantitative variables, just to express the difference between the two groups in terms of these variables. It was not significant, and in table number two, we brought a number of qualitative variables to compare the two groups, variables that we thought might have an effect on the score of awareness or ROSA, such as body mass index. But with this table, we have shown that there is no significant difference between the two groups.

Before the intervention, no significant difference was observed in the two groups in terms of quantitative demographic variables (Table 1).

**Table 1:** Distribution of demographic information (quantitative demographic variables).

Variable	Intervention group	Control group	*P*-value
	Mean ± SD	Mean ± SD	
Age	34.33 ± 5.18	34.43 ± 5.41	(*P* = 0.91)
Number of deliveries	1.10 ± 1.05	1.20 ± 1.05	(*P* = 0.60)
Work experience	7.61 ± 4.15	7.95 ± 4.32	(*P* = 0.66)
Hours of computer work per week	23.96 ± 2.82	24.03 ± 2.74	(*P* = 0.89)

The other demographic information was measured by the Chi-square test in the IG and CG. No statistically significant difference was observed between the two groups (Table 2).

**Table 2:** Distribution of demographic information (qualitative demographic variables .

Variable		Intervention group		Control group		*P*-value
		No.	%	No.	%	
Education level	Diploma	5	8.30	10	16.70	0.51
	Associate degree	14	23.30	14	23.30	
	B.A	31	51.70	25	41.70	
	M.A	10	16.70	11	17.50	
Total	120	60	100	60	100	
Marital status	Single	14	23.30	12	20	0.90
	Married	42	70	44	73.30	
	Divorced or widowed	4	6.70	4	6.70	
Total	120	60	100	60	100	
Body Mass Index	Underweight	9	15	8	13.30	0.97
	Healthy weight	25	41.70	27	45	
	Overweight	20	33.30	20	33.30	
	Obese	6	10	5	8.30	
Total	120	60	100	60	100	

The results of independent t test and paired t test showed Before the educational intervention, no statistically significant difference was found between the IG and CG in terms of ergonomic knowledge and ROSA. However, 6 months after the educational intervention, a significant increase was observed in ergonomic knowledge in the IG compared to the CG. The score of ROSA was reduced from a high-risk level to a low-and medium-risk level (Table 3). The risk of the final ROSA score is provided in (Table 4).

**Table 3:** Between-group comparison of mean ergonomic knowledge and ROSA scores before the intervention and 6 months later.

Variable	Group	Before the intervention mean ± SD	After the intervention mean ± SD	Significance level*
Ergonomic knowledge	Intervention (*n* = 60	7.20 ± 1.17	9.06 ± 0.63	<0.001
	Control (*n* = 60)	7.25 ± 1.27	7.18 ± 1.35	0.53
	Significance level**	0.82	<0.001	<0.001
ROSA	Intervention (*n* = 60)	6.01 ± 1.85	3.86 ± 1.47	
	Control (*n* = 60)	6.00 ± 1.14	5.95 ± 1.06	0.65
	Significance level**	0.93	<0.001	

* Paired *T*-test.

** Independent *T*-test.

**Table 4:** Distribution of risk level score according to ROSA before and after the intervention.

Variable	Before the intervention		After the intervention	
	Intervention group	Control group	Intervention group	Control group
ROSA	Frequency (%)	Frequency (%)	Frequency (%)	Frequency (%)
1	0 (0)	0 (0)	0 (0)	0 (0)
2	0 (0)	4 (6.70)	0 (0)	0 (0)
3	5 (3)	31 (51.70)	3 (5)	2 (3.3)
4	2 (3.3)	11 (18.30)	1 (1.70)	2 (3.3)
5	11 (18.30)	4 (6.70)	13 (21.70)	13 (21.70)
6	25 (41.70)	5 (8.30)	24 (40)	27 (45)
7	13 (21.70)	3 (5)	14 (23.30)	12 (20)
8	6 (10)	2 (3.3)	5 (8.30)	4(6.70)
Total	60	60	60	60

Before the intervention in the IG, 16 subjects (26.70%) were in the warning state (score between 3 and 5) meaning that they were in danger and if the same routine was continued, they would be exposed to skeletal injuries. Another 44 subjects (73.30%) needed ergonomic intervention (score above 5). After the intervention, 4 subjects (6.70%) were in a safe state, 46 subjects (76.70%) were in the warning state, and 10 subjects (16.70%) needed ergonomic intervention. In the CG, before and after the intervention, 17 subjects (28.30%) were in the warning state and 43 subjects (71.70%) needed ergonomic intervention.

According to the NMQ results, the frequency of pain in computer users’ different body parts is shown in Table 5. The highest frequency of pain in the IG and CG was related to the back (56.70% and 55%, respectively). The neck, shoulders, wrists, and elbows were next. Also, before the intervention, according to the chi-square test, no significant difference was found between the IG and CG in terms of the prevalence of musculoskeletal pain in different parts of the body. However, after the intervention, a significant difference was observed (Table 5).

**Table 5:** Distribution of musculoskeletal disorders in different parts of the body, before and after the intervention.

Variable		Before the intervention			After the intervention
		Intervention group	Control group	Intervention group	Control group
		Frequency (%)	Frequency (%)	Frequency (%)	Frequency (%)
Neck		32 (53.30)	31 (51.70)	17 (28.30)	31 (51.70)
	–	28 (46.70)	29 (48.30)	43 (71.70)	29 (48.300)
Significance level*		0.85		0.009	
Shoulder		31 (51.70)	30 (50)	14 (23.30)	28 (46.70)
	–	(48.30) 29	30 (50)	46 (76.70)	32 (53.30)
Significance level*		0.85		0.007	
Elbow		25 (41.70)	25 (41.70)	13 (21.70)	(45) 27
	–	35 (58.30)	35 (58.30)	47 (58.80)	(55) 33
Significance level*		0.999		0.007	
Wrist		29 (48.30)	28 (46.70)	10 (16.70)	28 (46.70)
	–	31 (51.70)	32 (53.30)	50 (83.30)	32 (53.30)
Significance level*		0.86		<0.001	
Back		26 (43.30)	27 (45)	5 (12.50)	28 (46.70)
	–	34 (56.70)	33 (55)	55 (91.70)	32 (53.30)
Significance level*		0.84		<0.001	
Lower back		34 (56.70)	33 (55)	13 (21.70)	32 (53.30)
	–	26 (43.30)	27 (45)	47 (87.30)	28 (46.70)
Significance level*		0.84		<0.001	
Hip		16 (26.70)	17 (28.30)	6 (10)	18 (30)
	–	44 (73.30)	43 (71.70)	54 (90)	42 (70)
Significance level*		0.83		0.006	
Knee		18 (30)	19 (31.70)	6 (10)	20 (33.30)
	–	42 (70)	41 (68.30)	54 (90)	40 (66.70)
Significance level*		0.84		0.002	
Ankle		10 (16.70)	10 (16.70)	3 (5)	11 (18.30)
	–	50 (83.30)	50(83.30)	57(95)	49(81.70)
Significance level*		0.999		0.02	

*Chi-square test.

Then we conducted the ANOVA test to check the effect of training in the intervention group compared to the control group, the results of which can be seen in Table 6.

**Table 6:** ANOVA test results between two intervention and control groups in the research variables.

Variable		Sum of squares	df	Mean square	F	Sig.
Rosa	Between Groups	72.200	1	72.200	14.181	.000
	Within Groups	37.750	78	.484		
	Total	109.950	79			
Neck	Between Groups	1.012	1	1.012	7.997	.006
	Within Groups	9.875	78	.127		
	Total	10.887	79			
Shoulder	Between Groups	.450	1	.450	3.162	.009
	Within Groups	11.100	78	.142		
	Total	11.550	79			
Elbow	Between Groups	2.113	1	2.113	13.646	.000
	Within Groups	12.075	78	.155		
	Total	14.188	79			
Wrist	Between Groups	2.813	1	2.813	17.308	.000
	Within Groups	12.675	78	.162		
	Total	15.487	79			
Back	Between Groups	2.113	1	2.113	14.614	.000
	Within Groups	11.275	78	.145		
	Total	13.388	79			
Lower Back	Between Groups	1.800	1	1.800	14.400	.000
	Within Groups	9.750	78	.125		
	Total	11.550	79			
Hipe	Between Groups	1.013	1	1.013	7.067	.010
	Within Groups	11.175	78	.143		
	Total	12.188	79			
Knee	Between Groups	1.250	1	1.250	8.442	.005
	Within Groups	11.550	78	.148		
	Total	12.800	79			
Ankle	Between Groups	2.113	1	2.113	.409	.024
	Within Groups	402.775	78	5.164		
	Total	404.887	79			

The results of the ANOVA test show that there is a significant difference between the intervention and control groups in all research variables.

## 4 Discussion

To encourage computer users to accept and observe correct ergonomic habits and correct body postures, it is essential to raise awareness in the workplace. Therefore, the present study intends to determine the effect of training through email correspondence on correct body postures among computer users at Birjand University of Medical Sciences.

The present findings showed that 6 months after the intervention, the ergonomic knowledge was increased in the IG compared to the CG. These results are consistent with a body of research, including the results reported by Sezgina et al. (35) and Mohammadi et al. (36) that showed promotion in ergonomic knowledge after an educational intervention. The educational intervention in the present study was through email correspondence. Studies on the usefulness of computer-assisted learning produced positive results such as increased knowledge and behavior of physical activity in female working staff (37, 38), and the emergence of ergonomic behaviors in preventing back pain in nurses (39). The results reported by Kishore et al. (10) and Jacob et al. (40) on training through web-based ergonomics showed improvement in the working staff's ergonomic knowledge.

The results of the present study showed that most musculoskeletal disorders in computer users were in the back, neck, shoulders, and wrists. Most musculoskeletal disorders in these parts of the body are due to repetitive tasks with low mobility. The repetitive tasks with low mobility have been considered as the risk factors involved in this type of musculoskeletal disorder (41, 42). Moeini et al. (43) showed that computer users mostly suffered from neck, back, shoulder, back, wrist, and knee injuries. They are at a higher risk of musculoskeletal disorders than other parts of the musculoskeletal system. In a study by Stanam et al., the prevalence of musculoskeletal symptoms in computer users in university staff was 60% at the waist, 58% at the neck, and 49% at the shoulders. This is consistent with the present study (44). In a body of research by Hashemi et al. (21), Habibi et al. (1), Moshki et al. (45), and Samadi et al. (46), the prevalence of musculoskeletal symptoms in computer users was higher in the back and neck than other parts, respectively. Mohammadipour et al. aimed to identify the prevalence of musculoskeletal disorders and ergonomic risks in the personnel of the Kerman University of Medical Sciences. These researchers showed that the highest prevalence of musculoskeletal disorders was in the back (72.4%) and neck (55.2%) (47). In general, work-related musculoskeletal disorders affect the soft tissues of the neck, back, shoulders, and hands (48). Similarly, Zayed et al. (49) reported that among computer users, the back was highly injured (56.5%) followed by the neck (51.5). The elbow was the least injured (18.5%) (49). Akrouf investigated the prevalence of musculoskeletal disorders in computer users in Kuwait with results showing that the prevalence of pain in the neck area was 53.5%, the back 51.1%, the shoulder 49.2%, and back 38.4% (50). In their study, Jensen et al. concluded that the prevalence of neck pain among female computer users is 53%, followed by shoulder pain at 42% and wrist pain at 30% (51). The results of the present study showed that after the educational intervention, the frequency of musculoskeletal disorders in the IG was significantly reduced compared to the CG. A significant change in the reduction of musculoskeletal disorders in the IG is mainly related to this increased knowledge of desired behavior, gaining positive experiences after learning methods that target the complications of musculoskeletal disorders, such as learning the correct way of sitting. The related literature shows that the knowledge of safe working conditions is helpful in severely reducing the complications of work-related musculoskeletal disorders (10). Studies by Esmaeilzadeh et al. (24), Norashikin et al. (52), and Baydur et al. (53) reported a reduction in musculoskeletal disorders in computer users of Istanbul Medical School, as well as the office staff of a company in Sydney and Turkish municipal personnel, respectively. In addition, other studies have shown that educational interventions are effective in reducing musculoskeletal disorders in the IG compared to the CG (46, 54). Educational interventions can affect how people behave at work and enable them to improve their ability to adapt to these environments, preventing or reducing the severity of musculoskeletal disorders at work by changing work behavior (55).

Based on the research findings of Bahrami et al., who investigated the effect of training on the frequency of pain recurrence in hospital employees, it showed that corrective exercises reduced the frequency of back pain recurrence and ergonomic interventions reduced the risk level in different organs (56). However, Kalantari's study showed that ergonomic recommendations do not affect the frequency of pain recurrence. The reason for this is probably the short duration of the intervention or the employees themselves failed to follow these recommendations, which is not in line with the results of the present study (57). The results of Moffett et al.'s research, which reviewed 390 randomized clinical trial articles from 1996 to 1999, showed that educational exercises were more effective than the treatment provided by general practitioners (58) and the group that underwent training significantly decreased the average pain intensity. decreased Returning to normal daily activities and returning to work are more reported in them.

In their review, Viljanen et al. consider the effect of training programs effective in reducing ergonomic problems caused by work (59). Rostami et al.'s study on training in reducing the potential of musculoskeletal disorders showed that proper training can improve improper posture (60) be effective. In the Habibi study, it was observed that the posture evaluation score in computer users decreased significantly after training (1). The results of our study are consistent with the research results of Bulduk et al. (61), Al-Qahtani et al. (62), Robertson et al. (63), Ghasemi (64) and Atari et al. (65).

The present findings showed that educational intervention managed to reduce computer users’ ROSA scores in the IG. 44 subjects (73.30%) who ​​needed ergonomic interventions (score above 5) were reduced to 10 subjects (16.70%) in need of ergonomic interventions. After implementing the intervention and training program, it was found that the user's knowledge increased compared to before the intervention, indicative of the computer users’ improved ergonomics while working at their workstations. It also shows that they are more likely to adjust the workstation, chair height, and other workplace accessories according to ergonomics. Thus, the inappropriate posture and, consequently, the ROSA score and its components were reduced. The related literature also reported a reduction in the mean ROSA score after educational interventions in the IG, which is consistent with the present study (45, 56, 66).

### 4.1 Limitation and analysis

One of the limitations of this study was the small number of participants. We suggest the sample size be increased in future studies of other researchers. Since checking e-mails is a daily task for employees, this E-learning training method was considered. However, some computer users may not see the full content of the email for reasons such as lack of time, which can be considered a limitation of this study. Another limitation of this study is the reliance on self-reporting participants, as well as comparing different types of training and content. Additionally, the obsolescence of some office equipment and the impossibility of replacing them for employees to improve ergonomic conditions are other limitations in achieving the expected results of the project. The strength of the present research is the presence of the study population in the workplace and the use of electronic content, which is cost-effective in terms of cost and time.

## 5 Conclusion

Training ergonomics through email correspondence is a practical and useful way to improve ergonomic behaviors among computer users. Training through E-learning enables improvement in the ability to adapt to the workplace and avoid skeletal disorders by applying ergonomic principles. By changing work behavior, occupational musculoskeletal disorders can be prevented, reducing risks and complications.

## Data Availability

The datasets used in the study are available from the corresponding author on reasonable request.
